# Occurrence of multiple mental health or substance use outcomes among bisexuals: a respondent-driven sampling study

**DOI:** 10.1186/s12889-016-3173-z

**Published:** 2016-06-10

**Authors:** Greta R. Bauer, Corey Flanders, Melissa A. MacLeod, Lori E. Ross

**Affiliations:** Epidemiology & Biostatistics, Schulich School of Medicine & Dentistry, Western University, K201 Kresge Building, London, ON N6A 5C1 Canada; Centre for Addiction and Mental Health, Toronto, ON Canada; Dalla Lana School of Public Health, University of Toronto, Toronto, ON Canada

**Keywords:** Sexual orientation, Mental health, Substance use, Epidemiology, Health inequalities

## Abstract

**Background:**

Bisexual populations have higher prevalence of depression, anxiety, suicidality and substance use than heterosexuals, and often than gay men or lesbians. The co-occurrence of multiple outcomes has rarely been studied.

**Methods:**

Data were collected from 405 bisexuals using respondent-driven sampling. Weighted analyses were conducted for 387 with outcome data. Multiple outcomes were defined as 3 or more of: depression, anxiety, suicide ideation, problematic alcohol use, or polysubstance use.

**Results:**

Among bisexuals, 19.0 % had multiple outcomes. We did not find variation in raw frequency of multiple outcomes across sociodemographic variables (e.g. gender, age). After adjustment, gender and sexual orientation identity were associated, with transgender women and those identifying as bisexual only more likely to have multiple outcomes. Social equity factors had a strong impact in both crude and adjusted analysis: controlling for other factors, high mental health/substance use burden was associated with greater discrimination (prevalence risk ratio (PRR) = 5.71; 95 % CI: 2.08, 15.63) and lower education (PRR = 2.41; 95 % CI: 1.06, 5.49), while higher income-to-needs ratio was protective (PRR = 0.44; 0.20, 1.00).

**Conclusions:**

Mental health and substance use outcomes with high prevalence among bisexuals frequently co-occurred. We find some support for the theory that these multiple outcomes represent a syndemic, defined as co-occurring and mutually reinforcing adverse outcomes driven by social inequity.

## Background

Frequency estimates of bisexual self-identification vary from 0.7 to 3.1 % of the population [1–3]. While bisexuals often are grouped with gay and lesbian participants in health research, or excluded [4], recent studies show bisexuals experience higher levels of mental health and substance use issues than their monosexual (i.e., attracted to only one gender) peers [5–7]. While findings of elevated risk may result from confounding induced by behavioural measures (requiring a minimum of two sex partners for bisexual classification) [8], differences have also been observed based on sexual orientation identity.

Bisexual-identified individuals generally report worse mental health and higher substance use than heterosexuals, including anxiety, depression, and negative affect [5], alcohol/drug use and/or suicidality [9–12], and tobacco use [13]. Studies have found similarities between gay and bisexual men, who tend to report worse mental health and more substance use than heterosexual men [11, 14–16]. Bisexual women often report worse mental health and suicidality than lesbians [9, 10].

While bisexual populations experience disparities in multiple individual mental health and substance use outcomes, it is unclear how often these outcomes co-occur within the same bisexuals, creating a high adversity burden and potential difficulties in resolution. This co-morbidity has, to our knowledge, been examined only once with regard to anxiety or mood disorder combined with heavy drinking, despite the fact that co-morbidity has important implications for service delivery to this population. In this study, 10.0 % of bisexual-identified people had the combined outcome, compared with 5.2 % of gay/lesbian people and 2.2 % of heteorsexuals [7]. Further, if a high burden of co-morbidity exists, it is unclear whether particular segments of bisexual populations bear a disproportionate risk and therefore should be targeted for intervention; this has not been explored in research previously. Heterogeneity between sub-groups of bisexuals is infrequently studied, as bisexuals are typically considered as one unified group in research (or even grouped with gay or lesbian participants). An intersectional framework for research – an approach that emerged from observations on the inability of research on race and (separately) gender to explain the intersecting impacts of racism and sexism on African-American women – emphasizes the importance of studying such experiences as health at different intersections, rather than treating categories as single unified groups [17–19]. In particular, an intracategorical complexity approach to intersectionality emphasizes the diversity of experience within larger master categories [17], and can be applied to bisexuality to better understand whether bisexual experience of health differ at intersections of other identities or social statuses.

The possibility of co-morbidity within a marginalized population also raises the question of syndemicity. While co-morbid conditions may have varying relationships, a syndemic is defined as synergistic epidemics within a population created through the mutual interaction and reinforcement of at least two health issues [20, 21]. Further, a syndemic is driven by inequity between and within populations, based on social class, age, gender, sexual orientation, and/or race or ethnicity [20–24]. Inequity can function to create a syndemic in multiple ways; for example, it can serve as a pathway in restricting access to resources and consistent care, or in can create stress, which then has a significant negative impact on health [21].

The current paper explores whether high frequencies of five mental health or substance use conditions (depression, anxiety, suicidal ideation, problematic alcohol use, and polysubstance use) co-occur in a bisexual population and assesses the level of burden of co-morbidity. The paper also explores whether heterogeneity exists in prevalence of multiple outcomes in different sociodemographic groups within bisexual population, and addresses the possibility of a syndemic.

## Methods

Methods were approved by the Research Ethics Board at the Centre for Addiction and Mental Health, Toronto, Canada. Participants indicated their consent to participate in the online survey after reading the letter of information, by clicking a button saying “I have read and understood the information on the web page, and agree to participate in this research survey”.

### Study sample

Participants (*n* = 405) were age 16 and over; identified as bisexual, and lived in Ontario, a province containing 38 % of Canada’s population [25]. Participants were instructed to consider eligibility regarding their bisexuality as follows: “Our definition of bisexual includes people attracted to more than one sex and/or gender. This may include those who self-identify as bisexual, pansexual, omnisexual, 2-spirited, fluid, queer, questioning, or who choose not to use an identity label.” Throughout this manuscript, we use the term “bisexual” in reference to this attraction based definition (that is, to refer to the entire sample regardless of specific self-identification) except where explicitly noted.

Participants were recruited to complete an English-language Internet survey using respondent-driven sampling, a method of chain-referral sampling [26]. Initial seed participants were members of our Community Advisory Committee, who were purposefully recruited to reflect diversity in age, ethnoracial background, and region of Ontario. A second round of seed participants was introduced mid-way through data collection, and included individuals who had contacted the research coordinator directly to express interest in participating, and whose characteristics addressed gaps in the participant characteristics at that time (e.g., regarding gender). Participants could recruit up to 10 additional participants; recruitment included nine waves beyond 18 original seed participants [6]. To account for non-randomness in social networks, participants’ network sizes were obtained and recruitment networks tracked for use in analysis. Those missing data for more than one of five outcome variables (*n* = 18) were excluded, for a total sample of 387.

### Measures

#### Outcome measures

Our goal was to ensure that the outcome variables investigated represented a problem that would be of clinical significance, such that co-occurring outcomes could be interpreted to reflect a high burden. For this reason, we chose more stringent indicators of our mental health and substance use outcomes wherever possible. *Depression* was assessed using the 9-item Patient Health Questionnaire’s Depression Scale (PHQ-9) [27], which measures symptoms over the past 2 weeks. Summed scale values could range from 0 to 27 (Cronbach’s α = 0.87 in our data). Scores ≥10 indicated symptoms consistent with major depressive disorder [28]. *Suicide ideation* was assessed using two items from the Canadian Community Health Survey (CCHS), Cycle 4.1 [29]. CCHS items were selected for this study in order to allow for comparison with Canadian population-based data. The included items queried: “Have you ever seriously considered committing suicide or your own life?”; and “Has this happened in the past 12 months?” Responses were forward-filled to provide past-year measures for the entire sample, since the primary outcome in this study pertained to recent (rather than lifetime) mental health/substance use outcomes. *Anxiety* was measured using the 5-item Overall Anxiety and Impairment Scale (OASIS) [30]. Summed responses could range from 0 to 25 (Cronbach’s α = 0.88 in our data). Scores ≥8 identified symptoms consistent with an anxiety disorder [31]. *Problem drinking* was assessed with the 3-item Alcohol Use Disorders Identification Test (AUDIT) [32], using the higher men’s cut-off of 5 to accommodate all sexes/genders, including trans participants for whom there is no established cut-off (possible range: 0-12). *Polysubstance use* was coded based on past-year use of two of more (non-prescribed) substances on a checklist: amphetamines, barbiturates, club drugs (e.g., ketamine), cocaine, crack cocaine, crystal meth, hallucinogens, inhaled drugs, opiates, or PCP.

For our main outcome, *multiple mental health and/or substance use outcomes*, we decided *a priori* that the presence of three or more of the five outcomes would constitute a sufficient co-morbidity burden to consider serious, given the potential complications in addressing these issues in the presence of others. As it is possible for two outcomes to represent manifestations of one condition (e.g., depression and suicidal ideation), three outcomes ensures the presence of at least two distinct conditions. For descriptive purposes we also created two additional measures, one a count of the total number of outcome conditions for each participant, and the other a categorization of each possible combination of outcomes among those with three or more.

#### Socio-demographic factors

*Age* was categorized into four groups. Four *gender* categories were coded from two survey questions, one asking participants whether they were assigned a male or female sex at birth, and the other a check-list of gender identity categories; the four categories were cisgender men (those assigned male at birth who currently identify as men), cisgender women (assigned female at birth and identifying as women), trans men or assigned-female-at-birth genderqueer persons (those assigned female at birth who now identify as either men or another non-female gender such as genderqueer), and trans women or assigned-male-at-birth genderqueer persons (those assigned male at birth who now identify as women or another non-male gender). For *ethnoracial background*, participants indicating Aboriginal or First Nations ethnicity were coded as Aboriginal. Remaining participants who indicated they were always or sometimes perceived as a person or colour were coded as non-Aboriginal racialized, and participants who indicated they were not perceived as a person of colour or were unsure were coded as non-Aboriginal, non-racialized. While all participants identified under the broad umbrella of bisexuality as part of the study’s inclusion criteria, individual identity labels varied; *self-identified sexual orientation* was coded from a multi-category check-all-that-apply checklist as: bisexual only, bisexual plus at least one other identity, and other identities only (primarily pansexual and queer). Since access to both bisexual community and social services is unique in metropolitan Toronto, *residence in Toronto* was coded based on postal code.

#### Social equity factors

Four *education* categories ranged from high school or less to some/completed graduate education. *Income-to-needs ratio* was estimated by dividing the midpoint for household income categories by the number of individuals supported, and partitioning the result into weighted quartiles. Every-day and major event discrimination were measured using the *Perceived Discrimination Scale,* scored to range from 0 to 208 [33], and divided into quartiles (Cronbach’s α = 0.86 in our data). We used the 17-item *Anti-Bisexual Experiences Scale* to measure biphobia [34]. Scale responses could sum to 17-102 (Cronbach’s α = 0.84 in our data), and were grouped into weighted quartiles. C*hildhood abuse* was coded based on self-report of physical or sexual abuse that had occurred prior to age 16.

### Statistical analysis

Data were analysed using SAS version 9.3 or SAS-callable SUDAAN version 11.0. Analyses were weighted to represent the networked population of bisexuals age 16 and over in Ontario. Sample weights were calculated as the inverse of each participant’s network size, rescaled to sum to the sample size [35]. Variances were adjusted for clustering by shared recruiter. Descriptive means and proportions were estimated, with 95 % confidence intervals, for individual and combined outcomes, and for sociodemographic and social equity measures. Pairwise associations between all components of the outcome were estimated using phi coefficients (ϕ) to provide evidence in support of, or against, the existence of a syndemic, in which factors must be mutually reinforcing.

To explore whether particular groups of bisexuals bear a higher burden of multiple mental health and/or substance use outcomes, prevalences of the three-or-more-conditions outcome were estimated for each sociodemographic subgroup. Sociodemographics were then combined in a logistic regression model (Model 1) to estimate adjusted prevalence risk ratios (aPRRs) using average marginal risks [36].

We explored the role of adverse social conditions, including education, income, discrimination, anti-bisexual experiences and childhood abuse. Prevalences of the outcome were estimated for each categorical group. Each social equity factor was then entered, first singly (Model Series 2), then jointly (Model 3), into logistic regression models adjusted for sociodemographic factors. Prevalence risk ratios were estimated using average marginal risks [36], and 95 % confidence intervals using Taylor series linearization. For Model 1 and Model 3, we estimated Nagelkerke R^2^.

## Results

Table 1 presents weighted frequencies for sociodemographics, social equity factors, and outcomes. All five outcomes were common, ranging in frequency from 18.4 % for past-year suicidal ideation to 30.6 % for anxiety (please refer to Table 1 for 95 % confidence intervals). Among bisexuals, 37.2 % experienced none of the outcomes and 19.0 % experienced three or more. The four most common combinations of outcomes (≥2 % of bisexuals in each) were depression, anxiety and suicide ideation; depression, anxiety and problem drinking; depression, problem drinking and polysubstance use; and depression, anxiety, suicide ideation and problem drinking. Table 2 presents weighted phi coefficients, and their *p*-values. Of the 10 outcome variable pairings, 7 were significantly associated (*p* < 0.05). Depression was associated with each of the other four outcomes, while anxiety was associated with all outcomes other than problem drinking. Suicide ideation was associated with each of the two other mental health outcomes, but not with either of the substance use outcomes. Problem drinking was associated with polysubstance use as well as depression, and polysubstance use was associated with all outcomes other than suicide ideation. Statistically significant associations were all positive associations, and appear weak to moderate in strength (phi ranging from 0.1054 to 0.5458).

**Table 1:** Weighted sociodemographics, social factors, and mental health and substance use outcomes among Ontario bisexuals age 16 and over (*n* = 387)

	n	%	95 % CI
Sociodemographics			
Age (*N* = 383)			
16-24	96	27.7	(19.0, 36.4)
25-34	168	43.9	(35.0, 52.7)
35-44	75	16.8	(10.8, 22.8)
45+	44	11.7	(3.8, 19.6)
Gender (*N* = 384)			
Cisgender woman	214	58.0	(48.5, 67.6)
Cisgender man	73	24.9	(16.4, 33.4)
Trans woman^a^	26	4.5	(1.3, 7.7)
Trans man^a^	71	12.6	(8.1, 17.0)
Ethnoracial background (*N* = 385)			
Aboriginal	37	7.6	(3.8, 11.3)
Non-Aboriginal racialized	59	15.7	(8.0, 23.4)
Non-Aboriginal non-racialized	289	76.7	(68.8, 84.6)
Sexual orientation self-identity (*N* = 387)			
Bisexual only	69	25.2	(17.4, 33.0)
Bisexual and other identity/ies^b^	167	38.7	(30.3, 47.1)
Other identity/ies only^b^	151	36.0	(28.1, 43.9)
Residence (*N* = 380)			
Metropolitan Toronto	180	53.2	(44.1, 62.4)
Ontario, not in Toronto	200	46.8	(37.6, 55.9)
Social equity factors			
Education (*N* = 384)			
High school or less	37	9.7	(4.8, 14.7)
≥ Some postsecondary school	114	29.9	(21.7, 38.0)
Bachelor’s degree	127	39.1	(30.8, 47.3)
≥ Some graduate education	106	21.3	(15.6, 27.1)
Income-to-needs ratio quartile (*N* = 376)			
≤ $12,500/person/yr	103	26.0	(18.8, 33.2)
> $12,500 to $23,333/person/yr	82	22.5	(14.7, 30.2)
> $23,333 to < $35,000/person/yr	80	22.5	(16.2, 28.9)
≥ $35,000/person/yr	111	29.0	(20.0, 38.0)
Perceived discrimination quartile (*N* = 387)			
0-3	80	25.3	(18.6, 33.9)
4-10	107	28.8	(21.7, 36.0)
11-18	90	21.8	(16.0, 27.5)
19-108	110	23.1	(16.9, 29.4)
Anti-bisexual experiences quartile (*N* = 383)			
31-49	82	27.6	(19.3, 35.9)
50-63	77	23.4	(15.6, 31.3)
64-85	100	24.5	(18.3, 30.8)
86-188	114	24.4	(17.9, 30.9)
Childhood abuse (*N* = 387)			
Yes	200	43.6	(36.9, 50.4)
No	187	56.4	(49.6, 63.1)
Mental health and substance use			
Depression (PHQ-9 ≥ 10)	114/383	29.7	(21.9, 37.6)
Anxiety (OASIS ≥ 8)	118/387	30.6	(22.8, 38.5)
Suicide ideation, past yr	83/387	18.4	(12.9, 24.0)
Problem drinking (AUDIT ≥ 5)	114/351	30.1	(22.2, 37.9)
Polysubstance use^c^	107/387	21.7	(15.6, 27.7)
Number of outcomes (*N* = 387)			
0	132	37.2	(29.4, 44.9)
1	91	25.3	(19.5, 31.0)
2	81	18.6	(13.3, 23.9)
3	55	13.3	(8.0, 18.6)
4	22	4.5	(1.8, 7.2)
5	6	1.2	(0.2, 2.3)
3 or more outcomes	83	19.0	(12.9, 25.1)
Outcome combinations (*N* = 387)			
<3 outcomes	304	81.0	(74.9, 87.1)
Depr. + Anx. + Suic.	19	4.6	(1.6, 7.6)
Depr. + Anx. + Drink.	6	2.5	(0.0, 5.3)
Depr. + Anx. + Polysub.	6	1.9	(0.0, 4.6)
Depr. + Suic. + Drink.	2	0.3	(0.0, 0.8)
Depr. + Suic. + Polysub.	1	0.1	(0.0, 0.2)
Depr. + Drink. + Polysub.	8	2.3	(0.4, 4.2)
Anx. + Suic. + Prob. Drink.	0	0	--
Anx. + Suic. + Polysub.	5	0.6	(0.0, 1.3)
Anx. + Drink. + Polysub.	6	0.9	(0.1, 1.8)
Suic. + Drink. + Polysub.	2	0.1	(0.0, 0.2)
Depr. + Anx. + Suic. + Drink.	7	2.0	(0.1, 4.0)
Depr. + Anx. + Suic. + Polysub.	7	0.9	(0.0, 2.1)
Depr. + Anx. + Drink. + Polysub.	5	0.7	(0.0, 1.4)
Depr. + Suic. + Drink. + Polysub.	3	0.9	(0.0, 1.9)
Anx. + Suic. + Drink. + Polysub.	0	0	--
All 5 outcomes	6	1.2	(0.2, 2.3)

^a^Includes those who identify as genderqueer, bigender, 2-Spirit or other non-male, non-female identities

^b^Examples of other identities included pansexual, lesbian, gay, queer, etc.

^c^Past-year use of two or more illicit substances for non-medical use, excluding marijuana

**Table 2:** Associations between individual mental health and substance use outcomes among Ontario bisexuals age 16 and over (*n* = 387)

	Depression φ *p*-value	Anxiety φ *p*-value	Suicide ideation φ *p*-value	Problem drinking φ *p*-value	Polysubstance use^a^ φ *p*-value
Depression (PHQ-9 ≥ 10)	1.0000 --	0.5458 <0.0001	0.3424 <0.0001	0.1226 0.0224	0.1078 0.0349
Anxiety (OASIS ≥ 8)		1.0000 --	0.2859 <0.0001	-0.0552 0.3054	0.1054 0.0392
Suicide ideation past yr			1.0000 --	-0.0148 0.7819	0.0711 0.1627
Problem drinking (AUDIT ≥ 5)				1.0000 --	0.3199 <0.0001
Polysubstance use^a^					1.0000 --

^a^Past-year use of two or more illicit substances for non-medical use, excluding marijuana

Table 3 presents frequencies of the outcome (≥3 of 5 mental health/substance use conditions) for each subgroup. Significant differences were found for income-to-needs ratio and perceived discrimination. Without controlling for other factors, people with more annual household income per person had a lower burden of multiple mental health and/or substance use outcomes (7.0 % of bisexuals in the top quartile versus 22.8 % in the bottom quartile). Those reporting lower levels of discrimination also had a lower burden of multiple outcomes: 6.6 % of bisexuals in the lowest discrimination quartile (0-3) versus 32.0 % in the highest quartile (19-108).

**Table 3:** Prevalence of high mental health and/or substance use outcome by subgroups: Ontario bisexuals age 16 and over (*n* = 387)

	Frequency, high burden outcome^a^		
	%	95 % CI	p*
Sociodemographics			
Age			0.8720
16-24	20.7	(9.2, 32.2)	
25-34	21.0	(10.4, 31.5)	
35-44	13.7	(2.9, 24.5)	
45+	18.0	(0.0, 38.6)	
Gender			0.3830
Cisgender woman	19.7	(10.8, 28.6)	
Cisgender man	12.3	(2.0, 22.6)	
Trans woman^b^	29.5	(5.1, 53.9)	
Trans man^b^	25.5	(11.8, 39.2)	
Ethnoracial background			0.5424
Aboriginal	13.6	(3.1, 24.2)	
Non-Aboriginal racialized	14.2	(1.5, 27.0)	
Non-Aboriginal non-racialized	20.3	(13.0, 27.7)	
Sexual orientation self-identity			0.1795
Bisexual only	24.7	(9.5, 39.9)	
Bisexual and other identity/ies^c^	12.4	(4.7, 20.2)	
Other identity/ies only^c^	22.1	(14.1, 30.0)	
Residence			0.9038
Metropolitan Toronto	18.8	(9.6, 27.9)	
Ontario, not in Toronto	19.5	(10.9, 28.2)	
Social equity factors			
Education			0.3372
High school or less	26.5	(6.4, 46.5)	
≥ Some postsecondary school	25.3	(11.8, 38.7)	
Bachelor’s degree	13.0	(3.0, 23.0)	
≥ Some graduate education	17.5	(8.0, 26.9)	
Income-to-needs ratio quartile			0.0478
≤ $12,500/person/yr	22.8	(10.5, 35.2)	
> $12,500 to $23,333/person/yr	18.1	(7.8, 28.4)	
> $23,333 to < $35,000/person/yr	27.6	(11.1, 44.1)	
≥ $35,000/person/yr	7.0	(0.9, 13.1)	
Perceived discrimination quartile			0.0038
0-3	6.6	(0.8, 12.4)	
4-10	23.8	(11.8, 35.8)	
11-18	13.8	(4.2, 23.3)	
19-108	32.0	(17.4, 46.7)	
Anti-bisexual experiences quartile			0.7651
31-49	15.2	(3.7, 26.6)	
50-63	19.5	(5.0, 34.0)	
64-85	18.2	(8.5, 27.8)	
86-188	24.3	(10.8, 37.8)	
Childhood abuse^d^			0.2970
Yes	22.5	(12.9, 32.2)	
No	16.3	(8.7, 23.8)	

*P-values are from Rao-Scott chi-square tests

^a^Having three of more of the following five outcomes: Current depression, current anxiety consistent with disorder, past-year suicidal ideation, problem drinking, or polysubstance use (use of two or more illicit substances excluding marijuana)

^b^Includes those who identify as genderqueer, bigender, 2-Spirit or other non-male, non-female identities

^c^Other identity options included: 2-spirited, ambisexual, asexual, biaffectionate, bisensual, fluid, heteroflexible, homoflexible, omnisexual, pansexual, queer, or other identities not specified

^d^Childhood abuse includes self-report of any sexual or physical abuse prior to age 16

Table 4 displays results of three sets of logistic regression models. Model 1 includes all sociodemographic factors. Model Series 2 includes each individual social equity variable, adjusted for sociodemographics. These PRRs test a potential effect of that variable on the outcome, holding non-modifiable sociodemographic factors constant. Model 3 includes all sociodemographic and social equity factors. Here, PRRs represent effects conditioned on all other factors in the model.

**Table 4:** Weighted model-adjusted prevalence risk ratios for sociodemographic and social equity factors on high mental health and/or substance use burden^a^ among Ontario bisexuals age 16 and over (*n* = 387)

	Model 1: Sociodemographics			Model Series 2: Individual social equity variables, adjusted for sociodemographics			Model 3: All social equity variables, adjusted for sociodemographics		
	aPRR	95 % CI	*p*	aPRR	95 % CI	*p*	aPRR	95 % CI	*p*
Non-modifiable Sociodemographic Factors									
Age			0.8175						0.4360
16-24	1.00	--					1.00	--	
25-34	0.98	(0.48, 2.00)					1.65	(0.76, 3.55)	
35-44	0.65	(0.25, 1.70)					1.73	(0.75, 3.96)	
45+	0.87	(0.17, 4.31)					2.15	(0.85, 5.43)	
Gender			0.0991						0.0455
Cisgender woman^b^	1.00	--					1.00	--	
Cisgender man^b^	0.58	(0.25, 1.38)					0.81	(0.42, 1.57)	
Trans woman^c^	2.12	(1.13, 3.96)					2.44	(1.35, 4.42)	
Trans man^c^	1.20	(0.65, 2.20)					1.16	(0.69, 1.94)	
Ethnoracial background			0.2757						0.2554
Aboriginal	0.51	(0.18, 1.44)					0.64	(0.27, 1.52)	
Non-Aboriginal racialized	0.57	(0.20, 1.61)					0.45	(0.13, 1.54)	
Non-Aboriginal non-racialized	1.00	--					1.00	--	
Sexual orientation self-identity			0.1575						0.0007
Bisexual only	1.00	--					1.00	--	
Bisexual and other identity/ies^d^	0.42	(0.18, 1.01)					0.31	(0.17, 0.59)	
Other identity/ies only^d^	0.77	(0.44, 1.35)					0.64	(0.38, 1.09)	
Residence			0.9239						0.6749
Metropolitan Toronto	1.00	--					1.00	--	
Ontario, not in Toronto	0.97	(0.47, 1.98)					0.89	(0.52, 1.54)	
Social Equity Factors									
Education						0.0690			0.0108
High school or less				2.13	(0.80, 5.70)		2.41	(1.06, 5.49)	
≥ Some postsecondary school				2.07	(0.92, 4.67)		2.21	(1.25, 3.88)	
Bachelor’s degree				0.86	(0.37, 2.01)		0.89	(0.45, 1.77)	
≥ Some graduate education				1.00	--		1.00	--	
Income-to-needs ratio quartile						0.0464			0.0251
≤ $12,500/person/yr				1.00	--		1.00	--	
> $12,500 to $23,333/person/yr				0.78	(0.38, 1.63)		1.02	(0.49, 2.10)	
> $23,333 to < $35,000/person/yr				1.05	(0.52, 2.15)		1.39	(0.84, 2.28)	
≥ $35,000/person/yr				0.28	(0.08, 0.94)		0.44	(0.20, 1.00)	
Perceived discrimination quartile						0.0098			0.0006
0-3				1.00	--		1.00	--	
4-10				3.51	(1.15, 10.74)		3.77	(1.22, 11.64)	
11-18				2.22	(0.69, 7.14)		1.96	(0.62, 6.25)	
19-108				5.40	(1.76, 16.58)		5.71	(2.08, 15.63)	
Anti-bisexual experiences quartile						0.5994			0.6918
31-49				1.00	--		1.00	--	
50-63				1.41	(0.62, 3.23)		1.07	(0.58, 1.96)	
64-85				0.95	(0.42, 2.16)		0.73	(0.38, 1.41)	
86-188				1.46	(0.58, 3.71)		1.08	(0.60, 1.97)	
Childhood abuse^d^						0.5680			0.9248
Yes				1.00	--		1.00	--	
No				1.23	(0.60, 2.50)		0.98	(0.62, 1.53)	

^a^Having three of more of the following five outcomes: Current depression, current anxiety consistent with disorder, past-year suicidal ideation, problem drinking, or polysubstance use (use of two or more illicit substances excluding marijuana)

^b^Cisgender includes those whose gender identities match the sex they were assigned at birth

^c^Includes those who identify as genderqueer, bigender, 2-Spirit or other non-male, non-female identities

^d^Other identity options included: 2-spirited, ambisexual, asexual, biaffectionate, bisensual, fluid, heteroflexible, homoflexible, omnisexual, pansexual, queer, or other identities not specified

^e^Childhood abuse includes self-report of any sexual or physical abuse prior to age 16

While no sociodemographic group experienced significantly higher raw frequencies of multiple outcomes (Table 3), significant effects emerged after adjusting for all other variables (Table 4) suggesting that some factors may potentially play causal roles and should be evaluated in longitudinal studies. In model 3, gender and sexual orientation identity were significant. Trans women were 2.44 times as likely to have multiple mental health and/or substance use outcomes as cisgender women (95 % CI: 1.35, 4.42). Within this group of broadly defined bisexuals, self-identified sexual orientation was associated with multiple outcomes (*p* = 0.0007). Compared with those who self-identified as bisexual only, those self-identifying as bisexual and another identity(ies) were one-third as likely, and those who self-identified as only other identities were two-thirds as likely, to have multiple outcomes.

Among social equity factors, education approached significance in model 2 and was significant in model 3. Controlling for all other factors, bisexuals with high school education or less were 2.41 times as likely to have multiple outcomes as those with at least some graduate education (95 % CI: 1.06, 5.49). Income-to-needs was again found to be significant in models 2 and 3. In model 2, controlling only for sociodemographics, people in the highest income quartile were 0.28 times as likely to have multiple outcomes as people in the lowest income quartile (95 % CI: 0.08, 0.94); after controlling for other social equity factors, they were 0.44 times as likely (95 % CI: 0.20, 1.00).

The final social equity factor associated with multiple outcomes was perceived discrimination. In models 2 and 3, people in the highest discrimination quartile were more likely to have multiple outcomes than people in the lowest quartile; significant effects emerged in the second-lowest quartile wherein even this group had elevated risk over the lowest quartile. This suggests that the higher frequencies of multiple outcomes indicated by those who report discrimination (frequency = 32 % among those in the highest discrimination quartile) may represent a causal effect of discrimination, as a strong effect persisted after controlling for sociodemographics and other social equity factors.

Neither the Anti-Bisexual Experiences Scale nor childhood abuse were associated with multiple outcomes in any part of our analysis. The pseudo-R^2^ of model 1 was 0.0911, indicating approximately 9 % of outcome variance was explained by sociodemographic factors. In model series 2, the pseudo-R^2^ varied for each model, and in model 3 it increased to 0.3628, indicating approximately 36 % of outcome variance was explained by sociodemographic and social equity factors. This suggests that variables included in the model explain a substantial amount, but not all of the variance in having three or more of these mental health and/or substance use outcomes.

## Discussion

While research indicates bisexual populations experience a high prevalence of individual mental health or substance use conditions [5, 6, 9–16], a focus on individual outcomes disallows a fuller examination of the larger burden of mental health and substance use in these populations. Our results demonstrate that these outcomes are often not independent, and a considerable proportion of bisexual people in Ontario (19.0 %) live with a substantial burden, indicated by the simultaneous presence of three or more outcomes. Unadjusted group comparisons are most useful for identifying real-life inequalities between groups, which may be useful in targeting areas of highest service needs. Here, those in the lower three income-to-needs quartiles were more likely to experience three or more outcomes, as were those in the upper three quartiles for perceived discrimination. No other factors were associated in crude comparisons. Given that bisexuals in our study were on average relatively young and more likely to be female – a demographic distribution that mirrors those from population-based samples [1, 3] – we note that age and gender were not associated with multiple outcomes in unadjusted analysis. This reflects a recent finding from an analysis of related outcomes in Canadians age 18 to 59; in this analysis for example, gender was not associated with co-occurring anxiety or mood disorder and heavy drinking, despite being associated with each of the outcomes individually [7]. This appears to be due to the different directions of effect for each outcome (females reporting higher prevalence of anxiety or mood disorders and males of problem drinking).

When associations were each adjusted for sociodemographic variables, the previous associations (with income and perceived discrimination) remained, and gender and education approached significance. After adjustment for all other factors, all four of these variables were significantly associated with the co-occurrence of multiple outcomes. The potential that these variables play causal roles in either initiating or maintaining multiple outcomes should be explored in future longitudinal research. Trans women’s elevated risk for having 3 or more outcomes is consistent with research that shows connections between social marginalization of trans people and mental health [37]. We note that our cross-sectional study only allowed the prediction of prevalent cases of multiple outcomes. Research on bisexuals has been limited, and future research collecting longitudinal data would be useful to differentiate between factors leading to incident outcomes versus factors that contribute to prolonged duration of multiple mental health or substance use outcomes, and to better ascertain temporality.

Interestingly, differences in sexual orientation self-identification impacted the risk of having multiple outcomes. Although more than 60 % of attraction-defined bisexuals (as per our inclusion criterion) indicated they self-identified with the label “bisexual”, a substantial proportion of attraction-defined bisexuals (36.0 %) did not choose the bisexual label at all on a sexual orientation self-identity item. We found that after adjusting for all other factors, those who self-identified as only bisexual were at greater risk for multiple mental health and/or substance use outcomes than those who self-identified as bisexual plus another identity(ies) or as only non-bisexual identities such as pansexual, 2-spirited, asexual, or queer. There is emerging evidence that bisexual-identified people report experiencing more discrimination than do non-monosexuals who use other self-identity labels; [38] it is possible that this differential exposure to discrimination may be associated with different risk for co-occurring outcomes. Future qualitative or mixed-methods research may help explore the significance of the identity labels people choose for themselves, and their relationship to mental health and substance use.

Our study establishes that the co-occurrence of multiple outcomes is prevalent among bisexual people. Considering the popularity of syndemic theory in the field of public health, and particularly as it pertains to sexual minority people [39], this raises the question of whether the observed multiple outcomes represent a syndemic. Research on syndemics in public health has not coalesced around a coherent conceptualization and corresponding methodology, and it is unclear that phenomena frequently identified as “syndemics” always differ from classical co-morbidity [40], or even simple morbidity; some analysis strategies use a summed variable for psychosocial conditions [39], and effects may thus be driven by changes from zero conditions to one. It is also unclear to what extent “mutual reinforcement” between outcomes is assessable; some work defines co-occurring outcomes to include those so temporally distant that causation could only occur unidirectionally (e.g., childhood abuse and later-life conditions). Some research implicitly positions social inequities as drivers of a syndemic, others as outcomes within a syndemic, and some position syndemic conditions as unidirectionally causing a single additional outcome.

As such, we take a conservative approach in our interpretation and conclude that mental health and/or substance use outcomes frequently co-occur among bisexuals, generating a high prevalence of both individual and multiple adverse outcomes. The positive pairwise associations between the individual outcomes we observed suggest this co-occurrence might be mutually reinforcing in some cases. Our findings support the larger syndemic hypothesis that multiple mental health and/or substance use outcomes in bisexual populations are driven by social conditions (a characteristic of syndemics), wherein lesser education, higher discrimination levels, and being a trans woman were associated with the presence of multiple outcomes, and being in the highest income-to-needs ratio quartile was protective. In our data, multiple outcomes represent a current (or recent, e.g. past-year) condition, whereas most of these factors are measured in ways that would have allow for existence over a longer timeframe. We do caution that temporality is not perfectly separated here; for example, some reported discrimination may have occurred as a result of mental health or substance use issues. Despite these limitations, our results are consistent with the idea of a syndemic of multiple mental health and/or substance use outcomes among bisexuals.

Our finding that bisexuals in the highest quartiles for perceived discrimination were at higher risk for co-occurring multiple mental health and/or substance use outcomes is consistent with previous research, proposing an association between experience of discrimination and mental health among bisexuals [41] and sexual minority people in general [42]. However, our finding that a measure of biphobia experiences (the Anti-Bisexual Experiences Scale) was not associated with our mental health/substance use outcome was unexpected. This may suggest important differences in health impact for different types of discrimination experiences. While the Perceived Discrimination Scale measures perceived interpersonal acts of discrimination (serious incidents and everyday discrimination) that could be associated with any number of stigmatized identities (e.g., being treated with less respect, being unfairly treated by the police, being unfairly fired or denied a promotion), the Anti-Bisexual Experiences Scale measures perceived interpersonal insults or assumptions that are specific to bisexuality and might be described as microaggressions (e.g., assumptions of promiscuity). Microaggressions are the sometimes unintentional commonplace behavioural or verbal indignities that communicate discriminatory messages through small but repeated insults, exclusions or attacks [43]. The PDS therefore captures a broader range of perceived discrimination experiences for a broader range of attributions. To explore the role of biphobia further, we ran a sensitivity analysis replacing the overall PDS with a value derived only from reported discrimination events attributed to participants’ bisexual orientation; it did not approach significance (results not shown). We note that the median score was 0, indicating that while bisexuals reported a range of bisexuality-related microaggressions, most reported no major biphobic discriminatory experiences. Thus, while discrimination impacted multiple outcomes among bisexuals, we did not find evidence to support that this was from biphobia-attributed discrimination or microaggressions.

Additional explanations for the substantial mental health and substance use burden among bisexuals remain to be determined. While childhood physical or sexual abuse was frequently reported, we did not find a significant effect on having multiple mental health and/or substance use outcomes in this bisexual population, which contrasts with results from general population research on individual outcomes [44]. This represents one important area for future investigation.

Although this study included a large sample of bisexuals, generalizability is limited as it was conducted using individual data from participants within one large province. While sexual minority persons face discrimination in Ontario, significant legal progress has been made to protect their civil rights. Canada decriminalized homosexuality in 1969, and provincial laws protecting against orientation-based workplace discrimination were instituted between 1977 and 1998. National marriage equality was attained in 2005. Because the policy context was similar for all participants, we were not able to examine policy impact at the group level. Further, social inequity was not measured in this investigation (e.g., structural inequity or violence), nor were individual factors (e.g., coping strategies) that could also affect one’s risk for the outcomes under study. Group-level factors could perhaps interact with individual-level experiences to modify their effects in ways that require investigation in future research. It is possible, for example, that anti-bisexual experiences may have different effects in contexts without human rights protections, and where discrimination is more likely to be accompanied by a material threat such as violence.

## Conclusions

Among bisexuals, 19.0 % had multiple (3 or more) mental health and/or substance use outcomes. We did not find variation in raw frequency of multiple outcomes across sociodemographic variables (e.g. gender, age). After adjustment, gender and sexual orientation identity were associated, with trans women and those self-identifying their sexual orientation as bisexual only more likely to have multiple outcomes. Social equity factors had a strong impact in both crude and adjusted analysis: controlling for other factors, high mental health/substance use burden was associated with greater discrimination and lower education, while higher income-to-needs ratio was protective.

The concept of syndemics has utility in understanding the confluence of multiple negative health outcomes, particularly as pertains to groups experiencing social inequity. However, despite growing use in public health, the concept lacks a consistently applied definition or corresponding analytical approach [40]. In our analysis of multiple mental health and/or substance use outcomes among bisexuals in Ontario, we attempt to advance understanding of this public health concept. Our finding that a substantial proportion of bisexuals report a high burden of multiple mental health and/or substance use outcomes also has clinical significance. Services and supports will be required that are prepared not only to address these co-morbidities, but also the unique social context of bisexual people, with regard to the social marginalization that appears to contribute to these outcomes.

## Abbreviations

AUDIT, Alcohol Use Disorders Identification Test; CCHS, Canadian Community Health Survey; OASIS, Overall Anxiety and Impairment Scale; PHQ-9, Patient Health Questionnaire’s Depression Scale; PRR, prevalence risk ratio
